# Effectiveness of an intelligent weight-bearing rehabilitation robot in enhancing recovery following anterior cruciate ligament reconstruction

**DOI:** 10.3389/fpubh.2025.1526105

**Published:** 2025-04-01

**Authors:** Yating Wen, Xinhong Wang, Yi Mao, Xiaolu Sun, Na Xu, Xiaoqing Han

**Affiliations:** Department of Spinal Degeneration and Oncology, Weifang People’s Hospital, Weifang, Shandong, China

**Keywords:** anterior cruciate ligament injury, anterior cruciate ligament reconstruction, robot, knee joint function, knee joint mobility, pain

## Abstract

**Aim:**

Orthopedic surgery patients frequently delay early rehabilitation due to postoperative discomfort. This is especially true for younger patients with anterior cruciate ligament injuries who are eager to return to sports after discharge. Despite the recognized benefits of early rehabilitation, a standardized protocol for determining safe weight-bearing timelines post-ACL reconstruction is lacking. This study aims to evaluate the effectiveness of an Intelligent Weight-Bearing Rehabilitation Robot in improving recovery outcomes for these patients.

**Design:**

A retrospective cohort study comparing outcomes between individuals who received the intervention and those in the control group.

**Methods:**

Ninety-two patients who underwent ACL reconstruction were chosen as subjects and separated into two groups: control and intervention, each with 46 patients, in the order of hospital admission. The control group got standard rehabilitation training, whereas the intervention group received rehabilitation training using the Intelligent Weight-Bearing Rehabilitation Robot. The intervention effects of both groups were compared.

**Results:**

The intervention group demonstrated significant improvements in knee joint function post-surgery compared to the control group. The mean range of motion (ROM) in the experimental group increased from 41.63 ± 5.97° pre-intervention to 55.89 ± 5.13° post-intervention, while the control group’s ROM improved from 40.65 ± 3.43° to 49.78 ± 5.27° (*t* = 5.635, *p* < 0.001). Similarly, the Health Status Score (HSS) increased from 43.07 ± 3.83 to 59.93 ± 3.30 in the experimental group, while the control group showed an increase from 43.76 ± 4.06 to 54.39 ± 4.39 (*t* = 6.850, *p* < 0.001). These findings indicate a more substantial recovery in knee joint functionality in the experimental group, suggesting that robotic-assisted rehabilitation facilitated enhanced functional recovery. Additionally, pain reduction was significantly better in the experimental group. At 24 h post-surgery, the Visual Analog Scale (VAS) pain score for the experimental group was 3.45 ± 0.96, compared to 3.98 ± 0.93 in the control group (*t* = −2.647, *p* = 0.010). At 48 h, the VAS score in the experimental group was 2.37 ± 0.49, significantly lower than the control group’s 3.09 ± 0.66 (*t* = −5.923, *p* < 0.001). By discharge, however, the difference in VAS scores between the two groups was no longer statistically significant (*p* = 0.096). Furthermore, the intervention group had a significantly shorter hospital stay (7.07 ± 0.83 days) compared to the control group (7.96 ± 1.01 days) (*t* = −4.630, *p* < 0.001). No complications, such as secondary fractures or deep vein thrombosis, were reported in either group during hospitalization.

**Conclusion:**

Utilizing the intelligent weight-bearing robot in post-ACL reconstruction rehabilitation significantly improves knee function, reduces discomfort, and shortens hospital stay, highlighting the importance of innovation in medical rehabilitation.

## 1 Introduction

The knee joint is highly complex, consisting of articulations between the femoral condyle, tibial plateau, and meniscal structures. This intricacy provides the knee joint with distinctive and resilient functioning, facilitating flexion, extension, internal and external rotation, along with anterior–posterior and medial-lateral motions. The knee joint comprises the medial and lateral tibiofemoral joints, as well as the patellofemoral joint. The medial and lateral condyles of the femur are convex, but the tibial plateau is flat, facilitating a combination of rolling and sliding movements of the femur on the tibia, with the menisci occupying the intercondylar gap (1, 2). From a morphological standpoint, the bone structure of the knee joint does not intrinsically restrict anterior–posterior or medial-lateral translation of the tibia. The stability of the knee joint predominantly depends on ligaments, with the anterior cruciate ligament (ACL) being the most essential (3–5). The ACL originates from the inner edge of the lateral femoral condyle and runs inward, forward, and downward before connecting to the front section of the intercondylar eminence of the tibia (6). It prevents anterior translation, external rotation, and overextension of the knee joint. ACL injuries are among the most prevalent and debilitating sports-related injuries, with over two million instances recorded worldwide each year, primarily affecting children and adolescents. Treatment options for ACL injuries include non-surgical treatments and surgical intervention, known as anterior cruciate ligament restoration (ACLR) (7–9).

Regardless of the treatment technique chosen, without a comprehensive rehabilitation program, ligament adhesions and reduced knee joint function may occur. Compared to traditional rehabilitation methods, lower-limb intelligent weight-bearing rehabilitation robots enable interactive human-machine engagement, providing more precise and controlled postoperative weight-bearing training (5, 10, 11). They provide patients with quantitative instructions, allowing them to see their walking power and gradually develop with scientific weight-bearing. Because of the specific nature of orthopedic surgery, patients frequently refuse early rehabilitation activities owing to postoperative pain. Furthermore, because ACL reconstruction patients are often younger than older adult patients undergoing joint replacement, there is a greater need for these patients to return to sports and physical activities. Despite the recognized benefits of early rehabilitation, a standardized protocol for determining safe weight-bearing timelines post-ACL reconstruction is lacking. Existing studies provide conflicting recommendations, with some supporting early ambulation to enhance recovery, while others caution against premature loading due to the risk of graft stress and muscle tension. This inconsistency highlights the need for more precise, evidence-based rehabilitation strategies. According to certain studies, early ambulation and weight-bearing activities after anesthesia recovery do not impair ACL graft healing and stability and may even minimize patellar discomfort and speed up recovery. Early weight-bearing activity, on the other hand, might cause skeletal muscle tension and increased ligament stress, which can impede recovery (11–13). Delayed ambulation and exercise due to intra-articular adhesions or ossification can cause muscle atrophy and weakening, compromising treatment results.

Moreover, the use of various rehabilitation robots has mostly been investigated in sectors such as stroke and hemiparesis (14–16). Given the lack of a standardized rehabilitation protocol, particularly regarding early weight-bearing training, it is essential to explore innovative rehabilitation technologies. Robotic-assisted rehabilitation has shown promising results in neurological recovery, yet its application in orthopedic rehabilitation remains underexplored. Therefore, this study investigates the effects of an Intelligent Weight-Bearing Rehabilitation Robot on post-ACL reconstruction recovery, aiming to determine its efficacy in improving functional outcomes and accelerating rehabilitation timelines. This report will detail the unique study procedure and findings.

## 2 Materials and methods

### 2.1 Research design and participants

A total of 100 patients who underwent ACLR from April 2020 to October 2022 at a tertiary hospital in Weifang were selected for our study population. Prior to the initiation of the study, patients will be briefed on the objectives and methodologies involved, as well as the possible advantages and risks associated with their participation. Consent was secured from the patient for the publication of the details in this case report. Individuals involved in the study have the option to exit at any point should they have enquiries or face any urgent circumstances throughout the investigation.

**Figure 1:**
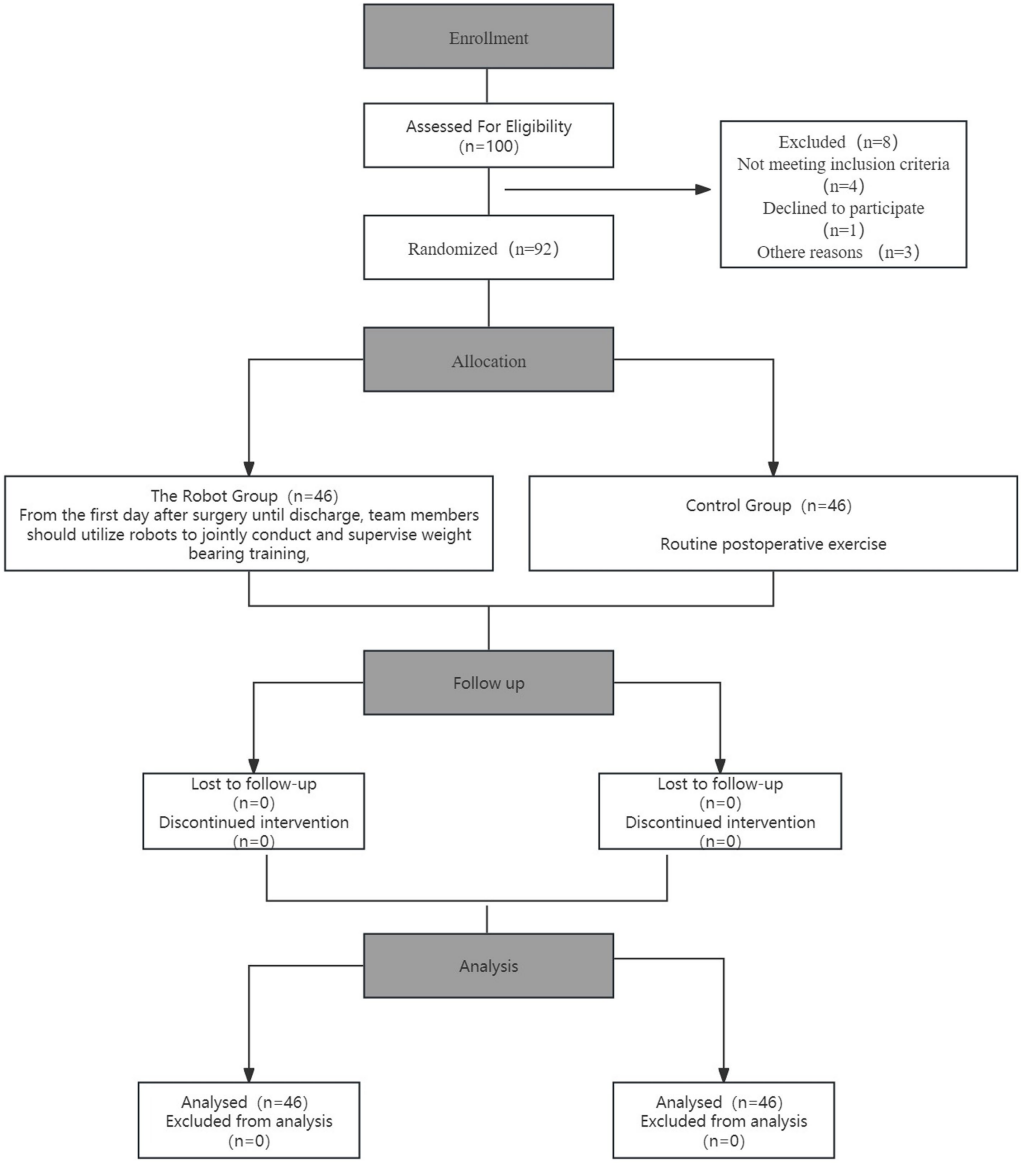
The workflow of the study.

The criteria for inclusion were established as follows: (a) participants aged between 18 and 45 years; (b) a confirmed ACL injury or rupture verified by MRI, all of whom had undergone ACLR surgery; (c) Participation in this study was voluntary for all patients. The criteria for exclusion encompassed the existence of concurrent meniscal injuries or fractures, significant osteoporosis or cartilage deterioration, deep vein thrombosis in the lower extremities, psychiatric disorders that impaired patient cooperation, and a heightened risk of postoperative infection. The control group included 50 patients hospitalized between April 2020 and April 2021, whereas the intervention group consisted of 50 patients hospitalized from May 2021 to October 2022. Throughout the intervention, 4 individuals in the intervention group were lost to follow-up, while 4 individuals in the control group were transferred to other hospitals for treatment. A total of 92 participants successfully finished the study. The timeline for enrolment, interventions, and assessments is detailed in Figure 1. Table 1 presents a comparison of general patient information for both groups. Further, to assess whether the achieved sample size was sufficient to detect a clinically meaningful difference, we conducted a post-hoc power analysis based on the primary outcome (HSS score improvement, ΔHSS). The resulting power was 0.9997, indicating sufficient power to detect the observed difference between groups. Detailed calculations are provided in Supplementary Materials. This investigation was carried out in full compliance with the Declaration of Helsinki and the protocols established by the local Ethical Review Board. Ethical approval was obtained from the Ethics Committee of REDACTED under protocol number (KYU20200401-4).

**Table 1:** Baseline characteristics of the included patients.

Item		Experimental group	Control group	*t/χ^2^*	*p*
Age		29.24 ± 6.94	29.04 ± 7.89	0.13	0.900
Gender	Male	36	34	0.24	0.625
	Female	10	12		
BMI		24.40 ± 2.20	23.96 ± 3.00	0.80	0.428
Operative side	Left knee joint	26	24	0.70	0.404
	Right knee joint	26	22		
Degree of trauma	Complete break	18	15	0.43	0.514
	Partial fracture	28	31		
Onset period	Acute stage	19	14	1.18	0.277
	Subacute stage	27	32		
Education	High school or below	4	3	4.74	0.192
	Undergraduate/college	36	39		
	Master degree or above	2	4		

All participants signed informed consent forms, which confirmed their understanding of the study details and their voluntary participation. Additionally, this study prioritized the confidentiality and anonymity of participant information and research data to safeguard the privacy rights of the individuals involved. All alterations to the study protocol, including adjustments in procedures, outcome measures, interventions, and data analysis, must be communicated to the Institutional Review Board in writing.

### 2.2 Study design

This study employed a retrospective cohort design, analyzing existing data to compare outcomes between individuals who received the intervention and those who did not. Participants were grouped based on their treatment history rather than through random assignment. The selection criteria for the experimental group (EG) and control group (CG) were predefined, and outcomes were assessed using statistical methods appropriate for observational studies.

### 2.3 Intervention

The team consisted of 12 individuals, including the Director of Orthopedics, two attending physicians, a rehabilitation therapist, the head nurse from the Orthopedics department, two nurses with expertise in Rehabilitation Medicine and Orthopedics, two engineers from the robotics manufacturer, a nursing graduate student. The group consisted of 7 males and 5 females, including 3 individuals with senior titles, 4 with intermediate titles, and 2 with junior titles. Furthermore, the team comprised 2 doctors, 6 individuals with master’s degrees, and 4 individuals with bachelor’s degrees. The Director of Orthopedics and the head nurse managed the administration and implementation of the rehabilitation program. Team members developed exercise regimens for different stages, drawing on clinical practice and referencing rehabilitation training programs from Peking University Third Hospital and the Shanghai Sixth Hospital’s Sports Medicine Research Institute. The department head, attending physicians, and rehabilitation therapist collaborated to discuss and create tailored postoperative rehabilitation exercise plans for each patient, taking into account their specific medical conditions. The head nurse conducted regular supervision and evaluation of the nursing procedures, maintaining quality control throughout the entire nursing process. Healthcare professionals from the orthopedics and rehabilitation medicine departments supported patients in learning how to utilize the intelligent weight-bearing rehabilitation robot. They outlined possible challenges encountered during application, delivered fundamental patient support, and adhered to medical guidelines for a range of procedures. The team from the robot manufacturer handled the adjustment of the parameters for the intelligent weight-bearing rehabilitation robot, as well as its installation, operation, maintenance, and servicing. The nursing graduate student engaged fully in the program’s implementation, taking on responsibilities for data collection, analysis, and organization.

### 2.4 Group 1 (the robot group)

We utilized the intelligent lower-limb weight-bearing rehabilitation robot developed by FaLuoShi (Shanghai) company. This device consists of three primary elements: an interface for human-machine interaction, a frame designed for walking assistance, and specialized rehabilitation footwear. Data transmission occurs via wireless Bluetooth technology. Prior to the utilization of the robot, patients received a comprehensive briefing regarding its intended purpose and operational methodology. Participants were guided to independently observe their heart rate, pulse, and the flexibility of the knee joint while using the device. Individuals were instructed to quickly communicate any atypical symptoms, including sounds from the knee joint, swelling, redness, acute pain, bleeding, or persistent discomfort. During the initial utilization of the weight-bearing robot by a patient, a diligent nurse facilitated the registration process. Upon entering their essential personal details and linking them with information regarding their physician and rehabilitation therapist, they were able to customize their rehabilitation training plan. Patients would subsequently link adjustable rehabilitation shoes and configure the weight-bearing parameters to commence the training.

**Figure 2:**
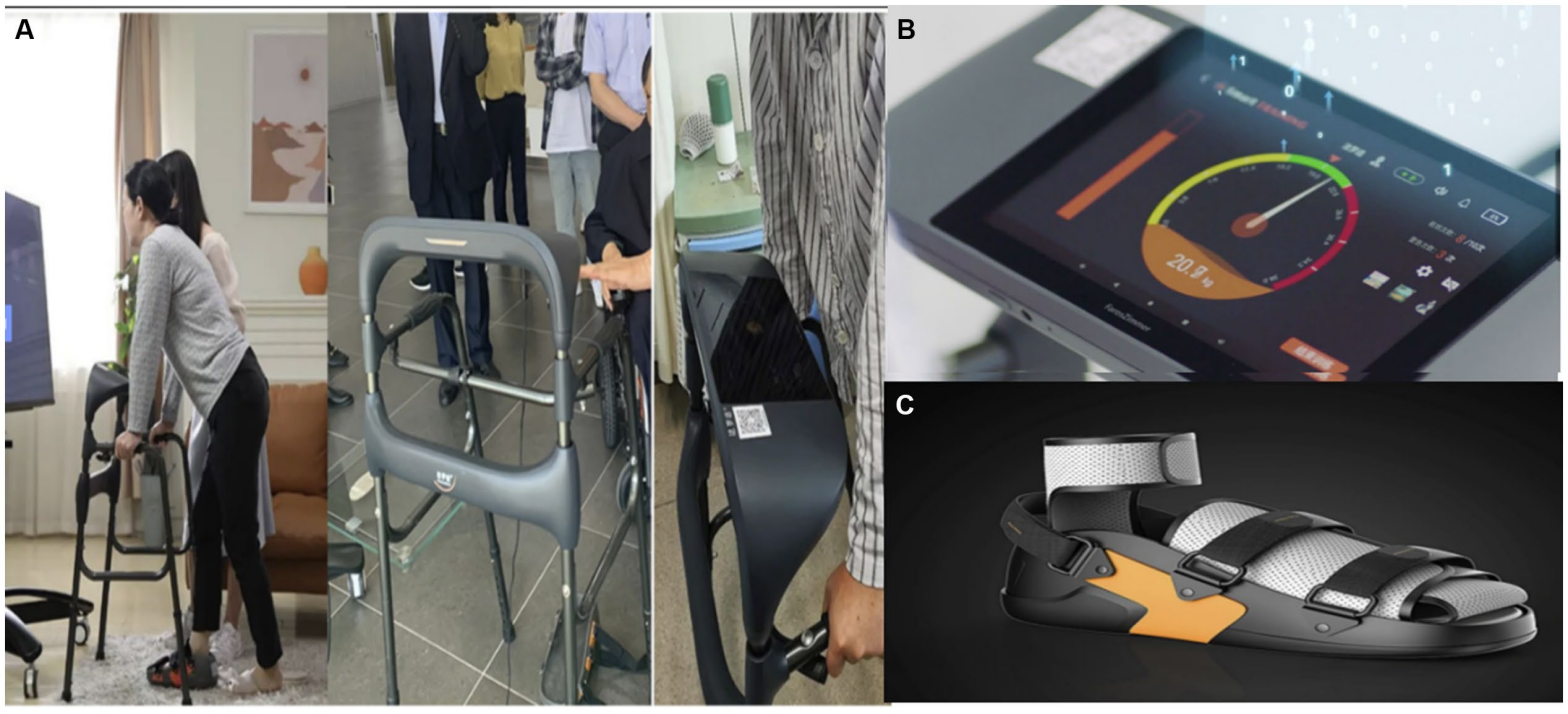
An intelligent wearable and visual knee joint weight-bearing exercise device. **(A)** The user wears the device and the subject exercises screenshots. **(B)** Visual weight-bearing exercise screens. **(C)** Rehabilitation shoes – adjustable size with built-in pressure sensor.

Prior to engaging in off-bed exercise, a systematic three-step process was implemented: lying down, sitting, and standing, with each position maintained for 30 s. On the first day following surgery, patients were permitted to stand on the ground for a duration of 3–5 min, with the support of medical personnel. Subsequently, if the patient experienced no discomfort, they would engage with the intelligent weight-bearing rehabilitation robot for partial weight-bearing walking. Patients were instructed to gently lean their torso forward, engage the hamstring muscles to alleviate the strain on the anterior cruciate ligament, and securely hold the walking aids on either side. The joint brace was positioned at a 0° flexion and extension lock. As the patient walked in the rehabilitation shoes, the indicator on the display adjusted in response to the predetermined weight-bearing level. When the pointer reached the green zone, it was recorded as one valid repetition. Should the pointer enter the red zone, appropriate notifications would alert the patient to refrain from applying excessive force. Throughout the robot’s operation, tailored modifications were implemented according to the patient’s condition on that particular day. The initial weight-bearing setting was limited to a maximum of one-third of the patient’s body weight. Following that, gradual increases in weight-bearing load varied between 15 and 30% of the patient’s body weight. The intervention occurred every 15–30 min, twice daily, until discharge, aiming to achieve a minimum of 50% body weight bearing on the affected knee by the discharge date. Daily step counts were not rigidly defined and were modified according to various factors, including the patient’s tolerance, pain levels, extent of limb swelling, and adherence to the exercise regimen. Data collected from patients after exercise was uploaded to a cloud platform, enabling attending physicians or rehabilitation therapists to assess the training plan and the actual completion level from the previous day prior to each exercise session. The data can be systematically monitored and assessed, encompassing exercise frequency, duration, and weight-bearing intensity. Individuals who failed to meet the established criteria received notifications. During patient exercise sessions, Canon music was utilized, with the volume tailored to align with individual patient preferences. The systematic approach to progressive weight-bearing relied on adhering to medical guidance, with the quadriceps engaging independently while managing the knee’s swelling and pain effectively (visual analog scale <5). This approach aligned with the principle of “integrated bone health.” The entire process was carried out with continuous oversight from attending physicians and rehabilitation therapists. Following each exercise session, cold compresses using ice packs were utilized to minimize swelling, as illustrated in Figure 2.

### 2.5 Group 2 (the control group)

The control group received conventional perioperative functional rehabilitation, which consisted of a series of standard physical therapy exercises and interventions designed to promote early recovery. These interventions were tailored to the individual needs of patients, with clinical assessments conducted by the medical team at regular intervals. The key components of the conventional rehabilitation program included: (a) Ankle Pumps: Performed daily, starting immediately post-surgery. Patients were instructed to perform 3 sets of 20 repetitions, 2–3 times per day. This exercise aimed to improve circulation and prevent deep vein thrombosis (DVT). (b) Straight Leg Raises: Initiated on the first postoperative day, with patients performing 3 sets of 15 repetitions per session. This exercise was done 2–3 times a day to enhance quadriceps activation and reduce muscle atrophy. (c) Weight-Bearing Activities: Gradual weight-bearing was encouraged, starting with partial weight-bearing on crutches. The intensity increased incrementally based on pain tolerance and physician recommendations, starting with 10–15 min per session, 3 times per day, and progressively advancing as tolerated. (d) Patients and their families received verbal instructions immediately after surgery, covering topics such as post-surgical care, pain management, and activity restrictions. Informational pamphlets were distributed to provide written guidance on exercises, expected recovery timelines, and self-care strategies. (e) Cold Packs: Applied for 20–30 min, 3–4 times a day, during the first 3 days post-surgery to control inflammation and reduce pain. Cold therapy was administered immediately after exercises to manage post-exercise discomfort. (f) Used 2–3 times a week for 15–20 min per session to promote soft tissue healing, reduce muscle spasms, and enhance circulation in the postoperative area. (g) Both groups underwent arthroscopic anterior cruciate ligament (ACL) reconstruction using autogenous hamstring tendon grafts. This procedure was performed by the same surgical team under general anesthesia to ensure consistency. The surgical technique, including graft harvest and placement, was identical for all participants. Post-operative care protocols were also standardized across both groups.

### 2.6 Outcome measures

A head nurse and a graduate student cross-checked and collected data both before and after the intervention (on the day of admission and discharge). Hospital for Special Surgery. Knee score (HSS): This scale has seven items: pain, joint function, flexion contracture, muscular strength, and joint stability, with a total score of 0 to 100. A higher score suggests improved knee joint function (17, 18).

Active joint range of motion (ROM): With the patient seated and relaxed, set the axis of a goniometer on the lateral femoral condyle. The fixed arm is put along the femur’s midline, parallel to the greater trochanter, and the measuring arm is placed along the fibula’s midline, aligned with the patient’s lateral malleoli. The measurement instrument is fastened to the afflicted limb, and the patient is told to fully flex and extend the knee joint in its normal condition. The final result is reported as the average of three measurements (19, 20).

The Visual Analog Scale (VAS) is one of the most widely used pain rating systems. Patients are given a 10 cm horizontal line on paper, with ‘0’ at one end and ‘10’ at the other. The middle section depicts various levels of suffering. Patients are instructed to note the intensity of discomfort they are feeling on the line, with no external interference. Scores below 3 indicate modest discomfort; scores between 4 and 6 suggest pain that interferes with sleep but is acceptable, while scores between 7 and 10 indicate terrible pain (21, 22).

A comparison of hospitalization days and the occurrence of complications (such as subsequent ligament damage, joint effusion, deep vein thrombosis in the lower leg, etc.) between the two groups.

### 2.7 Data analysis

The results of this experiment were analyzed using SPSS 23.0. Descriptive statistics for categorical data were reported as frequencies (percentages), while continuous data were expressed as (*x ± s*), with ‘*x*’ representing the mean and ‘*s*’ denoting the standard deviation. Statistical analysis consisted of independent sample t-tests, chi-squared tests, Wilcoxon rank-sum tests, and repeated measures analysis of variance (ANOVA). We used an analysis of covariance (ANCOVA) to discover the factors impacting the outcome indicators. This statistical approach enabled us to account for the impacts of certain covariates, which are continuous factors that may influence the dependent variable. By integrating these covariates into the model, we hoped to isolate the effects of the independent variables of interest on the outcome indicators while accounting for any pre-existing disparities that may be attributable to the covariates. The significance level for hypothesis testing was set at *α* = 0.05. In this study, *p* < 0.05 was regarded as statistically significant. This study has been prepared in accordance with the STROBE guidelines for observational studies (Table S1).

## 3 Results

### 3.1 The general characteristics of participants

The mean age of the experimental group was 29.24 ± 6.94 years, while the control group had a mean age of 29.04 ± 7.89 years, with no statistically significant difference between the two groups (*p* = 0.900).

Regarding gender distribution, 36 participants (78.3%) in the experimental group were male, and 10 (21.7%) were female. In the control group, 34 participants (73.9%) were male, and 12 (26.1%) were female. The difference in gender distribution between the two groups was not statistically significant (*p* = 0.625).

Body mass index (BMI) also showed no significant differences between the experimental group (24.40 ± 2.20) and the control group (23.96 ± 3.00) (*p* = 0.428). Other demographic and clinical variables, including the operative side, degree of trauma, onset period, and education level, showed no significant differences between the two groups (all *p* > 0.05). These results confirm that the two groups were well-matched at baseline, providing a solid foundation for subsequent analyses. As shown in Table 1, the detailed results are presented.

### 3.2 Comparison of knee joint ROM and HSS before and after intervention between two groups

Prior to the intervention, no notable differences in range of motion or health status scores were observed between the two groups (*p* > 0.05). Before the intervention, there were no significant differences in range of motion or health status scores between the two groups (*p* < 0.05), confirming the comparability of the two groups at baseline. Specifically, the mean ROM was 41.63 ± 5.97° in the experimental group and 40.65 ± 3.43° in the control group (*t* = 0.946, *p* = 0.338). Similarly, the baseline HSS scores were 43.07 ± 3.83 in the experimental group and 43.76 ± 4.06 in the control group (*t* = −0.846, *p* = 0.400).

Following the intervention, both the ROM and HSS scores improved significantly in both groups, with the experimental group demonstrating more pronounced improvements. The post-intervention ROM increased to 55.89 ± 5.13° in the experimental group and to 49.78 ± 5.27° in the control group, with a statistically significant between-group difference (*t* = 5.635, *p* < 0.001). Similarly, the HSS scores after the intervention reached 59.93 ± 3.30 in the experimental group and 54.39 ± 4.39 in the control group, again showing a significant advantage in the experimental group (*t* = 6.850, *p* < 0.001).

**Figure 3:**
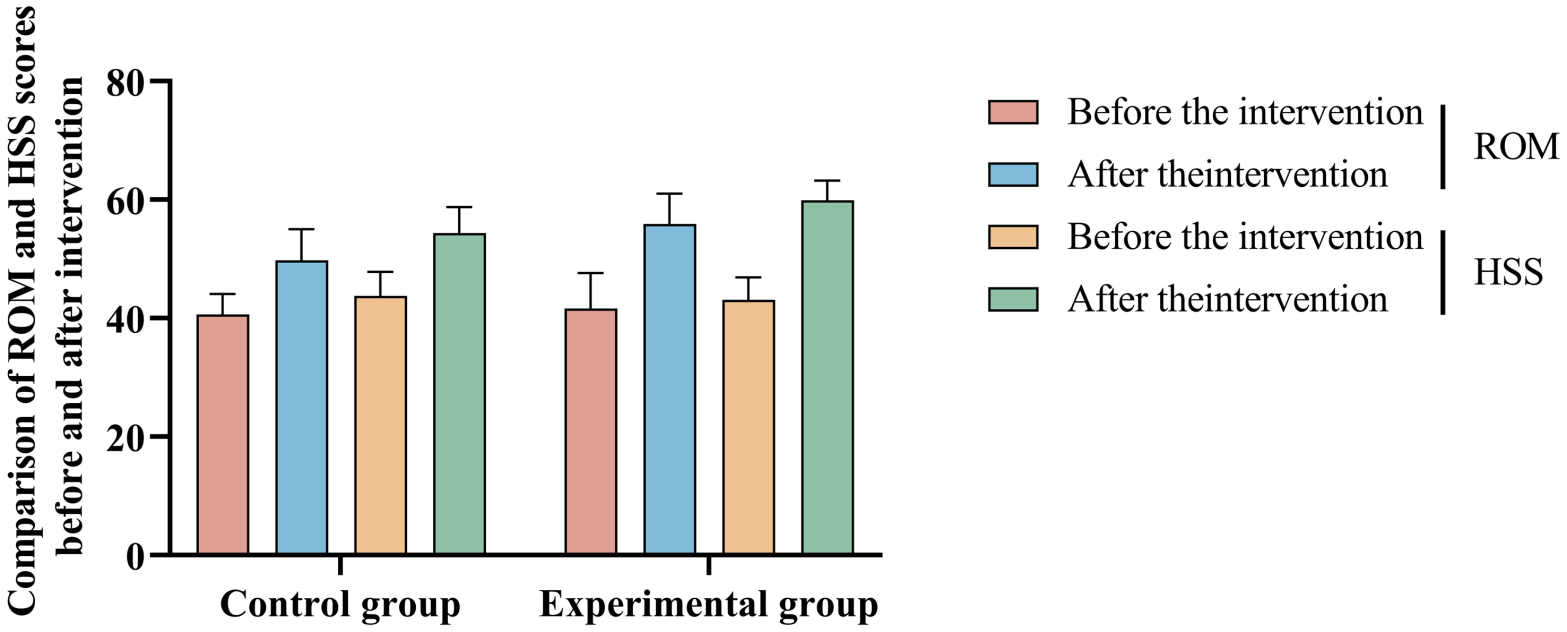
Comparison of knee joint ROM and HSS before and after intervention between two groups.

These findings indicate that the application of Lower Limb Weight-Bearing Rehabilitation Robots significantly improved knee joint mobility and function. The increase in ROM and HSS scores suggests that robotic-assisted rehabilitation facilitated more effective neuromuscular activation and progressive weight-bearing training, contributing to enhanced functional recovery. The more substantial gains in both ROM and HSS scores suggest that the robotic intervention facilitated better recovery and rehabilitation outcomes. This evidence supports the therapeutic potential of robotic-assisted rehabilitation in enhancing joint mobility and overall functional status. As shown in Table 2 and Figure 3, the detailed results are presented, with the latter offering additional graphical representation.

**Table 2:** Comparison of ROM and HSS scores before and after intervention.

	Control group	Experimental group	*t*	*p*
ROM (°)				
Before the intervention	40.65 ± 3.43	41.63 ± 5.97	0.95	0.338
After the intervention	49.78 ± 5.27	55.89 ± 5.13	5.64	<0.001
Difference	9.13 ± 5.61	14.26 ± 8.42	3.44	<0.001
HSS				
Before the intervention	43.76 ± 4.06	43.07 ± 3.83	−0.85	0.400
After the intervention	54.39 ± 4.39	59.93 ± 3.30	6.85	<0.001
Difference	10.63 ± 5.58	16.87 ± 4.88	5.71	<0.001

### 3.3 Comparison of VAS in different time periods between the two groups

There was no significant difference in Visual Analog Scale (VAS) pain scores between the two groups on the day of hospitalization (1.80 ± 0.78 vs. 1.85 ± 0.76; *t* = −0.271, *p* = 0.787), indicating comparable baseline pain levels. However, 24 h after surgery, the VAS scores of the experimental group were significantly lower than those of the control group (3.45 ± 0.96 vs. 3.98 ± 0.93; *t* = −2.647, *p* = 0.010), demonstrating a significant reduction in postoperative pain in the experimental group.

This trend continued 48 h post-surgery, with the experimental group reporting significantly lower VAS scores compared to the control group (2.37 ± 0.49 vs. 3.09 ± 0.66; *t* = −5.923, *p* < 0.001). These findings suggest that the use of robotic-assisted rehabilitation may have contributed to enhanced pain modulation by promoting early mobilization and reducing muscle stiffness, which are key factors in postoperative pain management. The trend of decreasing VAS scores over time, particularly within the first 48 h, highlights the potential of robotic interventions in mitigating early postoperative discomfort, leading to improved patient compliance and adherence to rehabilitation protocols.

By the day of discharge, however, the difference in VAS scores between the two groups was no longer statistically significant (1.91 ± 0.26 vs. 2.02 ± 0.33; *t* = −1.683, *p* = 0.096), indicating that pain levels had stabilized by the end of hospitalization for both groups.

**Figure 4:**
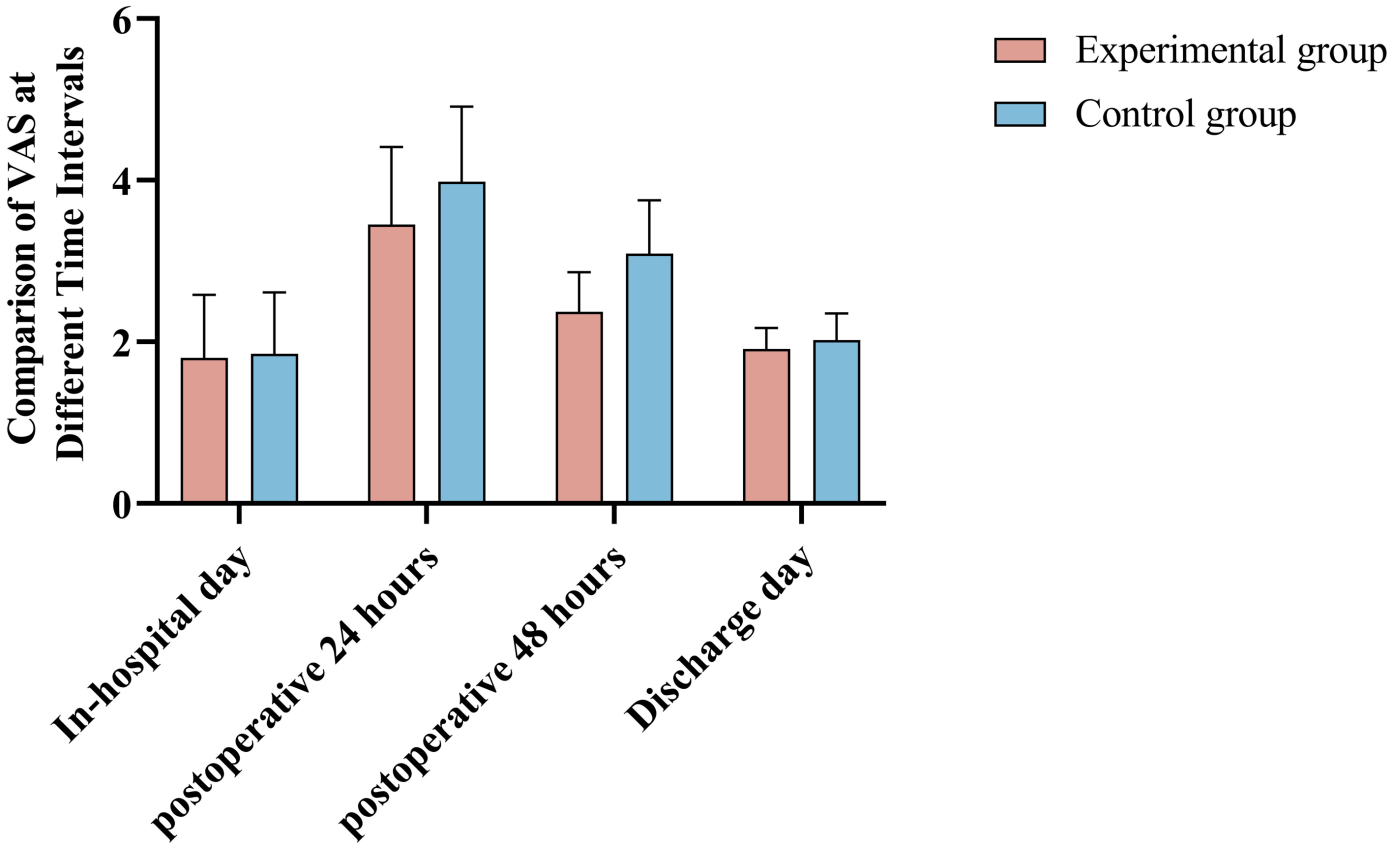
Comparison of VAS in different time periods between the two groups.

The repeated measures ANOVA showed significant effects for time (*F* = 138.46, *p* < 0.001), group (*F* = 26.674, *p* < 0.001), and the interaction between time and group (*F* = 4.86, *p* < 0.001), further confirming the impact of the intervention on postoperative pain reduction over time. As shown in Table 3, the detailed results are presented, with additional visual representation provided in Figure 4.

**Table 3:** Comparison of pain scores at two different time points.

	In-hospital day	Postoperative 24 h	Postoperative 48 h	Discharge day
Experimental group	1.80 ± 0.78	3.45 ± 0.96	2.37 ± 0.49	1.91 ± 0.26
Control group	1.85 ± 0.76	3.98 ± 0.93	3.09 ± 0.66	2.02 ± 0.33
*t*	−0.27	−2.65	−5.92	−1.68
*p*	0.787	0.010	<0.001	0.096

*F_time_ = 138.46, *F*_between groups_ = 26.674, *F*_interaction_ = 4.86, all *p* < 0.001.

### 3.4 Comparison of length of stay and incidence of complications between the two groups

The intervention group had an average hospitalization length of (7.07 ± 0.83) days, while the control group had an average of (7.96 ± 1.01) days, *t* = −4.630, *p* < 0. 001. During hospitalization, neither group exhibited secondary fractures of ligaments, joint effusion, or deep venous thrombosis in the lower limbs.

### 3.5 The subgroup analysis between the two groups

In this study, we utilized ANCOVA to assess the impact of gender, age, BMI, and education level on the postoperative rehabilitation results of patients who underwent ACL reconstruction with the aid of a lower limb weight-bearing rehabilitation robot. Analyses of subgroups were performed considering these demographic variables. The findings demonstrated that the effectiveness of the reconstruction, assessed through ROM and HSS, was not notably affected by any of these factors. The data indicated non-significant *p*-values (*p* > 0.05) across all examined groups, implying that the rehabilitative benefits of the lower limb weight-bearing rehabilitation robot were uniform irrespective of the patient’s gender, age, BMI, or education level. The comprehensive findings are presented in Table 4.

**Table 4:** Effects of the robot group as compared with the control group on the primary efficacy outcome in prespecified subgroups.

Subgroup	No. of patients (robot)	ROM (robot)	HSS (robot)	No. of patients (control)	ROM (control)	HSS (control)
Sex						
Male	36	55.86 ± 4.91	59.75 ± 3.52	34	49.39 ± 5.12	54.39 ± 4.78
Female	10	56.00 ± 6.15	60.60 ± 2.37	12	50.77 ± 5.72	54.38 ± 3.36
*p*		0.380	0.450		0.400	0.976
Education						
High school or below	6	53.75 ± 2.50	58.00 ± 2.16	2	52.50 ± 10.61	54.00 ± 4.24
Undergraduate/college	70	56.46 ± 5.28	60.06 ± 3.45	35	49.29 ± 5.02	54.71 ± 4.62
Master’s degree or above	16	54.29 ± 5.35	60.43 ± 2.99	9	51.11 ± 5.46	53.22 ± 3.63
*p*		0.174	0.446		0.723	0.653
BMI (kg/m2)						
<18.5	NA	NA	NA	2	45.00 ± 0.15	52.50 ± 3.54
18.5–23.9	16	56.36 ± 4.73	60.63 ± 3.28	21	50.00 ± 4.47	55.29 ± 4.63
>24	30	55.53 ± 5.37	59.57 ± 3.3	23	50.00 ± 6.03	53.74 ± 4.22
*p*		0.51	0.35		0.459	0.467
Age (years)						
≤35	37	56.24 ± 5.62	60 ± 3.44	38	49.61 ± 5.25	54.13 ± 4.38
>35	9	54.44 ± 1.67	59.67 ± 2.78	8	50.63 ± 5.63	55.63 ± 4.53
*p*		0.259	0.556		0.502	0.508

## 4 Discussion

This study represents a pioneering effort to investigate the use of an intelligent weight-bearing rehabilitation system following anterior cruciate ligament reconstruction. Applying weight to your knee promptly and appropriately following ACLR surgery promotes cartilage nourishment, aids in collagen recombination during the healing process, and restores the knee to its typical physiological load. Nonetheless, numerous patients with ACL injuries encounter postponed weight-bearing after surgery, attributed to pain, hesitance to apply pressure on the operated leg while standing, and challenges in exerting the appropriate force, resulting in swelling of the injured limb. These issues frequently persist throughout the typically lengthy clinical rehabilitation process (23, 24).

The intelligent weight-bearing rehabilitation robot for the lower limb enhances early knee joint function in ACL patients, leading to improved patient adherence and confidence in rehabilitation. This contributes to a reduction in hospital stay duration, as observed in our study. The findings indicated that the group receiving the intervention demonstrated superior HSS scores and range of motion when contrasted with the control group, along with reduced lengths of hospital stay. This can be linked to the intervention group beginning early weight-bearing on the first day after surgery, aided by the intelligent weight-bearing rehabilitation robot. Rehabilitation therapists and attending physicians collaboratively determined the approach and schedule for weight-bearing, following the principle of gradual, personalized exercise. The robot features integrated stress and gait sensors that assist patients in exercising according to predetermined weight-bearing levels. This method substitutes conventional subjective assessment, ungrounded training, and an absence of measurable criteria (25, 26). During training with rehabilitation shoes, patients receive prompts on force levels for each part as they stand and take steps, along with bidirectional feedback from the screen. In the event of discrepancies in force points, weight-bearing levels, or gait errors, the system promptly identifies and presents relevant red warning notifications. This primarily aids individuals in quadriceps training, minimizing shear force between the tibia and femur through the simultaneous contraction of the hamstring and quadriceps muscles, thus enhancing the strength of the muscles surrounding the knee joint. The daily target training plan and the actual training plan are communicated through Bluetooth technology, facilitating efficient monitoring and documentation of alterations in biomechanics and physiology throughout the complete rehabilitation training process. Attending physicians and rehabilitation therapists are capable of delivering quantitative evaluations rooted in patients’ daily rehabilitation advancements, thereby presenting authentic and dependable treatment strategies for future discharge recommendations. The analysis revealed no statistically significant difference in the incidence of complications between the two groups. This may be attributed to the advancing maturity of arthroscopic knee joint techniques, the surgical choices made by attending physicians, and the relatively brief duration of the interventions.

The intelligent weight-bearing rehabilitation robot for the lower limb has the potential to reduce postoperative pain in patients with ACL injuries. For individuals who experience significant discomfort, modifications in the intensity of weight-bearing activities and the application of cold compresses can be implemented promptly. Additionally, the robot assesses the extent of human functional recovery by analyzing data like patient muscle strength and gait, commencing progressive and passive partial weight-bearing activities right from the first day after surgery. The activities encompass partial weight-bearing standing training, sensory training while in a partial weight-bearing standing position, and gait training. These practices contribute to exercising the quadriceps, enhancing blood circulation, nourishing the transparent cartilage surrounding the patella, and preserving subchondral bone strength to a degree, thereby alleviating knee joint pain. In this experiment, rehabilitation therapists and attending physicians conducted personalized assessments tailored to each patient’s unique endurance and intraoperative conditions (21, 25, 27). Following the assessment results and discussions with the patient about their prior injury history and health concerns, a progressive functional training program was created. The program was systematically measured using specific indicators, and various weight-bearing guidance forces were chosen according to the patient’s lower limb mobility (28, 29). Should the force or position stray from optimal settings, the system offered relevant guidance and prompts. Patients reported improved comprehension, effectively tackling earlier challenges like vague explanations, lack of specificity, and brief training periods that lacked adequate intensity. The difference in pain scores between the two groups on the day of discharge was not statistically significant, likely due to the comprehensive, multimodal pain management strategies employed during hospitalization under the Enhanced Recovery After Surgery (ERAS) protocol, as well as the gradual recovery of knee function over time. Additionally, the use of the intelligent lower-limb weight-bearing rehabilitation robot facilitated assessment of functional recovery based on patient-specific metrics such as muscle strength and gait. Starting on the first postoperative day, patients engaged in progressive, passive partial weight-bearing activities, including partial weight-bearing standing exercises, sensory training in a partial weight-bearing standing position, and gait training. These targeted exercises contributed to early improvement in knee function and alleviated pain to some extent.

## 5 Conclusion

The use of lower limb intelligent weight-bearing robots in rehabilitation for patients undergoing anterior cruciate ligament reconstruction significantly improves knee joint function and enhances patients’ ability to perform daily activities. Additionally, the rehabilitation effect is consistent across various demographic factors, including age, gender, and BMI. Future research could explore optimizing the robot’s functionalities for different patient populations and further investigate its long-term effects on recovery outcomes.

## 6 Limitations

First, the limitations of the study include a limited sample size, a concentration of patients in a single hospital, and the lack of long-term follow-up and surveillance. Furthermore, the modification of specific parameters during installation requires refining, which will be further used and refined in clinical practice. Second, as a retrospective cohort study, there is a potential for selection bias due to non-randomized patient allocation. Although efforts were made to ensure comparability between groups, residual confounding variables such as preoperative functional differences, pain tolerance levels, and psychological factors may have influenced rehabilitation outcomes. Although we employed analysis of covariance (ANCOVA) to adjust for key baseline variables and minimize the influence of known confounders, residual confounding due to unmeasured or unrecognized factors cannot be entirely ruled out. Future multi-center studies with larger sample sizes are warranted to further validate these findings and improve generalizability.

## Data Availability

The original contributions presented in the study are included in the article/Supplementary material, further inquiries can be directed to the corresponding author.
