# Supplementary material for: Effectiveness of an intelligent weight-bearing rehabilitation robot in enhancing recovery following anterior cruciate ligament reconstruction
Source: Front Public Health. 2025 Apr 1;13:1526105. doi: 10.3389/fpubh.2025.1526105 (PMC11996826; doi:10.3389/fpubh.2025.1526105)
Supplement: Supplementary file 1 [file Data_Sheet_1.docx]

**Supplementary Materials**

**Post-hoc power analysis**

As this study is a retrospective cohort study, the sample size was determined by the total number of patients who met the inclusion and exclusion criteria within the specified study period (April 2020 to October 2022) at our institution. Nevertheless, to address your concern regarding whether the current sample size provides adequate statistical power, we further conducted a post-hoc power analysis based on the primary outcome (HSS score).

The effect size (Cohen’s d) was calculated using the following formula:

$$d=\frac{\bar{X}_{1}-\bar{X}_{2}}{\sqrt{\frac{({SD}_{1}^{2}+{SD}_{2}^{2})}{2}}}$$

where $\bar{X}_{1}$ and $\bar{X}_{2}$ represent the mean ΔHSS for the robot and control groups, ${SD}_{1}$ and ${SD}_{2}$ denote the standard deviations, respectively. Based on the observed data (ΔHSS mean: 16.87 vs. 10.63; standard deviation: 4.88 vs. 5.58), the calculated Cohen’s d was 1.19, indicating a large effect size. Using G*Power 3.1 software, we performed a post-hoc power analysis with the following parameters: a two-tailed independent samples t-test, α = 0.05, sample size of 46 patients per group, and an observed effect size (Cohen’s d) of 1.19. The achieved statistical power was 0.9997, indicating that the current sample size provides more than sufficient power to detect a clinically meaningful difference between groups.

Table S1. STROBE checklist of recommended items for observational studies.

| Item No. | Section | Recommendation | Relevant text from the manuscript |
| --- | --- | --- | --- |
| 1 | TITLE and ABSTRACT | / | / |
|  | a) | Indicate the study’s design with a commonly used term in the title or the abstract | Effectiveness of an Intelligent Weight-Bearing Rehabilitation Robot in Enhancing Recovery Following Anterior Cruciate Ligament Reconstruction. |
|  | b) | Provide in the abstract an informative and balanced summary of what was done and what was found | This study assessed a robotic intervention’s impact on anterior cruciate ligament (ACL) recovery, described the comparison of two groups (intervention vs. control), and summarized key findings: improved knee function, reduced pain, and shorter hospital stays in the intervention group. |
|  | INTRODUCTION | / | / |
| 2 | Background/rationale | Explain the scientific background and rationale for the investigation being reported | Orthopedic surgery patients frequently delay early rehabilitation due to postoperative discomfort. This is especially true for younger patients with anterior cruciate ligament injuries who are eager to return to sports after discharge. Despite the recognized benefits of early rehabilitation, a standardized protocol for determining safe weight-bearing timelines post-ACL reconstruction is lacking. |
| 3 | Objectives | State specific objectives, including any prespecified hypotheses | This study investigated the effects of an intelligent weight-bearing rehabilitation robot on post-ACL reconstruction recovery, aiming to determine its efficacy in improving functional outcomes and accelerating rehabilitation timelines. |
|  | METHODS |  |  |
| 4 | Study design | Present key elements of study design early in the paper | This study employed a retrospective cohort design, analyzing existing data to compare outcomes between individuals who received robotic-assisted rehabilitation and those receiving standard care. |
| 5 | Setting | Describe the setting, locations, and relevant dates, including periods of recruitment, exposure, follow-up, and data collection | Conducted at a tertiary hospital in Weifang, the study included patients undergoing ACL reconstruction from April 2020 to October 2022, with the control group from April 2020 to April 2021 and the intervention group from May 2021 to October 2022. Data were collected from admission to discharge. |
| 6 | Participants |  |  |
|  | a) | *Cohort study*—Give the eligibility criteria, and the sources and methods of selection of participants. Describe methods of follow-up | Patients aged 18–45 with MRI-confirmed ACL injuries who underwent ACLR and volunteered were included. Exclusions involved meniscal injuries, fractures, or conditions like osteoporosis. Among 100 initial participants, 92 completed the study after losses to follow-up or transfers. |
|  |  | *Case-control study*—Give the eligibility criteria, and the sources and methods of case ascertainment and control selection. Give the rationale for the choice of cases and controls | Not applicable. |
|  |  | *Cross-sectional study*—Give the eligibility criteria, and the sources and methods of selection of participants | Not applicable. |
|  | b) | *Cohort study*—For matched studies, give matching criteria and number of exposed and unexposed | Not applicable. |
|  |  | *Case-control study*—For matched studies, give matching criteria and the number of controls per case | Not applicable. |
| 7 | Variables | Clearly define all outcomes, exposures, predictors, potential confounders, and effect modifiers. Give diagnostic criteria, if applicable | Outcomes include knee function (HSS), range of motion (ROM), pain (VAS), hospital stay duration, and complications. The exposure is robotic vs. standard rehabilitation, with confounders like age, gender, and BMI considered. |
| 8* | Data sources/  measurement | For each variable of interest, give sources of data and details of methods of assessment (measurement). Describe comparability of assessment methods if there is more than one group | Data were collected by a nurse and graduate student at admission and discharge using standardized tools: HSS for knee function, goniometer for ROM, and VAS for pain. Methods were consistent across both groups. |
| 9 | Bias | Describe any efforts to address potential sources of bias | The study acknowledged selection bias risks due to non-randomized allocation in its retrospective design and notes efforts to ensure group comparability, though residual confounding (e.g., preoperative differences) may remain. |
| 10 | Study size | Explain how the study size was arrived at | The study started with 100 patients selected based on ACLR records from the specified period, resulting in 92 participants after accounting for losses, divided equally into two groups of 46. |
| 11 | Quantitative variables | Explain how quantitative variables were handled in the analyses. If applicable, describe which groupings were chosen and why | Quantitative outcomes (i.e., ROM, HSS, VAS) were analyzed as continuous variables, reported as means with standard deviations, without additional categorization. |
| 12 | Statistical methods |  |  |
|  | a) | Describe all statistical methods, including those used to control for confounding | Analysis involved t-tests, chi-squared tests, Wilcoxon rank-sum tests, and repeated measures ANOVA. ANCOVA was used to adjust for confounders like age, gender, BMI, and education level. |
|  | b) | Describe any methods used to examine subgroups and interactions | Subgroup analyses via ANCOVA explored effects of gender, age, BMI, and education on outcomes, assessing whether these factors modified the intervention’s impact. |
|  | c) | Explain how missing data were addressed | Participants with incomplete data due to loss to follow-up (4 per group) were excluded, leaving 92 participants with full data for analysis. |
|  | d) | *Cohort study*—If applicable, explain how loss to follow-up was addressed | Not applicable. |
|  |  | *Case-control study*—If applicable, explain how matching of cases and controls was addressed | Not applicable. |
|  |  | *Cross-sectional study*—If applicable, describe analytical methods taking account of sampling strategy | Not applicable. |
|  | e) | Describe any sensitivity analyses | No specific sensitivity analyses were conducted; however, baseline comparability was assessed to support robustness. |
|  | RESULTS | / | / |
| 13* | Participants |  |  |
|  | a) | Report numbers of individuals at each stage of study—eg numbers potentially eligible, examined for eligibility, confirmed eligible, included in the study, completing follow-up, and analysed | Initially, 100 patients were eligible; after exclusions and losses (8 participants), 92 were analyzed in our study, with 46 in each group (intervention and control). |
|  | b) | Give reasons for non-participation at each stage | Eight participants did not complete the study: 4 from the intervention group were lost to follow-up, and 4 from the control group were transferred to other hospitals. |
|  | c) | Consider use of a flow diagram | The flow diagram can be found in Figure 1. |
| 14* | Descriptive data |  |  |
|  | a) | Give characteristics of study participants (eg demographic, clinical, social) and information on exposures and potential confounders | Participants had comparable baseline characteristics. The mean age of the experimental group was 29.24 ± 6.94 years, while the control group had a mean age of 29.04 ± 7.89 years. 36 participants (78.3%) in the experimental group were male, and 10 (21.7%) were female. In the control group, 34 participants (73.9%) were male, and 12 (26.1%) were female. Other demographic and clinical variables, including the operative side, degree of trauma, onset period, and education level, showed no significant differences between the two groups (Table 1). |
|  | b) | Indicate number of participants with missing data for each variable of interest | Not applicable. |
|  | c) | *Cohort study*—Summarise follow-up time (eg, average and total amount) | Follow-up spanned from surgery to discharge, averaging 7.07 days (intervention) and 7.96 days (control), reflecting hospital stay duration. |
| 15* | Outcome data | *Cohort study*—Report numbers of outcome events or summary measures over time  *Case-control study—*Report numbers in each exposure category, or summary measures of exposure  *Cross-sectional study—*Report numbers of outcome events or summary measures | Intervention group showed greater improvements: ROM increased from 41.63° to 55.89°, HSS from 43.07 to 59.93; control group from 40.65° to 49.78° (ROM) and 43.76 to 54.39 (HSS). Pain (VAS) and hospital stay data also reported. |
| 16 | Main results |  |  |
|  | a) | Give unadjusted estimates and, if applicable, confounder-adjusted estimates and their precision (eg, 95% confidence interval). Make clear which confounders were adjusted for and why they were included | Significant between-group differences post-intervention: ROM (t=5.635, *P*<0.001), HSS (t=6.850, *P*<0.001), VAS at 24h (*P*=0.010) and 48h (*P*<0.001), hospital stay (t=-4.630, *P*<0.001). ANCOVA adjusted for age, gender, BMI, and education. |
|  | b) | Report category boundaries when continuous variables were categorized | Not applicable. |
|  | c) | If relevant, consider translating estimates of relative risk into absolute risk for a meaningful time period | Not applicable. |
| 17 | Other analyses | Report other analyses done—eg analyses of subgroups and interactions, and sensitivity analyses | Subgroup analysis via ANCOVA showed consistent intervention effects across gender, age, BMI, and education (*P*>0.05), indicating no significant modifiers (Table 4). |
|  | DISCUSSION | / | / |
| 18 | Key results | Summarise key results with reference to study objectives | The intervention group exhibited better knee function (HSS, ROM), reduced pain (VAS), and shorter hospital stays. |
| 19 | Limitations | Discuss limitations of the study, taking into account sources of potential bias or imprecision. Discuss both direction and magnitude of any potential bias | Limitations included a small sample size, single-center setting, lack of long-term follow-up, and potential selection bias from non-randomized design. |
| 20 | Interpretation | Give a cautious overall interpretation of results considering objectives, limitations, multiplicity of analyses, results from similar studies, and other relevant evidence | The robot enhances early recovery by improving knee function and reducing pain. Future research could explore optimizing the robot’s functionalities for different patient populations. |
| 21 | Generalizability | Discuss the generalisability (external validity) of the study results | Findings may be limited to similar settings and populations due to single-center design. Consistency across demographics suggests broad potential within this population. |
|  | OTHER INFORMATION | / | / |
| 22 | Funding | Give the source of funding and the role of the funders for the present study and, if applicable, for the original study on which the present article is based | / |
